# Quality of life and its influencing factors in patients with neuromyelitis optica spectrum disorder

**DOI:** 10.3389/fneur.2026.1797747

**Published:** 2026-06-19

**Authors:** Zhi-Yin Lei, Hui-Ping Li, Qing Yang, Xiao-Pei Zhang, Li-Xin Wang, Zhu-Yun Liu

**Affiliations:** 1 The Second Clinical College of Guangzhou University of Chinese Medicine, Guangzhou, Guangdong, China; 2 Department of Neurology, Guangdong Provincial Hospital of Chinese Medicine, Guangzhou, China; 3 State Key Laboratory of Traditional Chinese Medicine Syndrome, Guangzhou, China

**Keywords:** depression, neuromyelitis optica spectrum disorder, psychological resilience, quality of life, social avoidance, social support

## Abstract

**Background:**

Neuromyelitis optica spectrum disorder (NMOSD) imposes a significant burden on health-related quality of life (HRQoL). However, comprehensive studies examining HRQoL and its biopsychosocial determinants in Chinese NMOSD patients remain scarce.

**Methods:**

In this cross-sectional study, 216 Chinese patients with NMOSD underwent clinical evaluation for neurological disability (EDSS) and completed a battery of standardized self-report measures. These scales assessed HRQoL (SF-36), basic activities of daily living (BADL), depressive symptoms (PHQ-9), anxiety (GAD-7), sleep quality (PSQI), psychological resilience (CD-RISC), perceived social support (PSSS), general alienation (GAS), and social avoidance and distress (SAD). Multiple stepwise linear regression analyses were employed to identify independent associated factors of the Physical (PCS) and Mental (MCS) Component Summaries of the SF-36.

**Results:**

The cohort (median age 37, 85.2% female) showed significantly reduced median PCS (55.5) and MCS (51.0), with role-physical (25.0) and role-emotional (33.3) most impaired. In multivariate analysis, older age (*β* = −0.194), depressive symptoms (PHQ-9; *β* = −0.290), social avoidance (SAD; *β* = −0.128), and poor sleep (PSQI; *β* = −0.282) independently associated worse PCS (adjusted *R*^2^ = 0.402). For MCS (adjusted *R*^2^ = 0.576), depressive symptoms (*β* = −0.439), social avoidance (*β* = −0.135), poor sleep quality (*β* = −0.267), and lower resilience (CD-RISC; *β* = 0.127) were significant associated factors. (all *p* < 0.05).

**Conclusion:**

Psychosocial factors critically influence HRQoL in Chinese NMOSD patients. Multidimensional, culturally sensitive interventions targeting mental health, sleep, and social functioning may provide insights for improving patient outcomes.

## 1 Introduction

Neuromyelitis optica spectrum disorder (NMOSD) is an autoimmune disease of the central nervous system (CNS) mediated primarily by anti-aquaporin-4 (AQP4) antibodies, with primary pathogenic targeting of astrocytes (1). Its core clinical manifestations include recurrent optic neuritis, longitudinally extensive transverse myelitis, and area postrema syndrome, and it is a relapsing–remitting disorder associated with a high risk of cumulative disability.

The global prevalence of NMOSD is approximately 0.5 to 10 per 100,000 persons, with notable geographical and racial variations (2). The age-standardized prevalence in Asia is approximately 3.0 per 100,000, which is higher than that in Caucasian populations (approximately 1.0 per 100,000) (3). A striking female predominance is observed, with a female-to-male ratio as high as 8-9:1. Although NMOSD can occur at any age, peak onset typically strikes during early adulthood (median age 32–41 years) (3)—a period critical for career development, family responsibilities, and social engagement—thus rendering the disease impact particularly profound (4).

NMOSD is characterized by a high relapse rate and high disability burden. While biological agents have substantially reduced the annual relapse rate, each acute attack can lead to stepwise cumulative neurological damage (5). Historically, without adequate treatment, many patients rapidly developed permanent impairments, including severe visual loss, mobility limitations, chronic neuropathic pain, and sphincter dysfunction (6). Within 5 years of disease onset, approximately 50% of patients required wheelchair assistance and 30% became functionally blind (7). These severe physical impairments substantially compromise activities of daily living—such as dressing, bathing, and eating—as well as social and occupational participation, thereby significantly diminishing quality of life (QoL).

Health-related quality of life (HRQoL) is a multidimensional construct reflecting an individual’s subjective perception and satisfaction with their physical health, psychological state, level of independence, social relationships, and personal beliefs within their environmental context. Within the modern “bio-psycho-social” model of medicine, HRQoL has emerged as a core outcome measure in chronic disease management, parallel in importance comparable to traditional clinical metrics such as disability scores and neuroimaging (8). In NMOSD, impaired HRQoL is not an isolated concern but is interrelated with a cascade of adverse outcomes. Studies consistently show that NMOSD patients have significantly lower HRQoL scores than the general population and even worse than those with other chronic neurological conditions such as multiple sclerosis (MS) (9). This decline in QoL can trigger or exacerbate psychological disorders, particularly anxiety and depression, creating a vicious cycle of “physical illness–psychological distress–QoL deterioration”. Concurrently, impaired social functioning—including job loss, social withdrawal, and restricted family roles—undermines patients’ self-worth and life satisfaction (10).

The mechanisms driving HRQoL impairment in NMOSD are multifactorial. Disease-related factors such as greater disability, frequent relapses, severe fatigue, and chronic pain are primary contributors to reduced physical domain QoL (11–13). Psychologically, depression and anxiety are among the most prevalent comorbidities; they exert the strongest negative influence on mental domain QoL and may amplify the distress associated with physical disability (14). The point prevalence of major depressive disorder ranges from 29 to 46%, and Generalized Anxiety Disorder ranges from 20 to 37%, both significantly higher than in matched MS cohorts (15). Socioeconomically, fluctuating disability often necessitates occupational adjustments or withdrawal from work, with unemployment rates approaching 60% within 10 years of diagnosis (16). Furthermore, stigma, general alienation, and social avoidance frequently erode patients’ social support networks, while protective psychological traits like resilience are heavily taxed. Strong social support and high psychological resilience have been identified as crucial buffers against distress, whereas sleep disturbances and cognitive decline are increasingly recognized as hidden determinants of diminished well-being (17–19).

Despite mounting evidence confirming profound HRQoL impairment in NMOSD, significant knowledge gaps remain. Most existing studies have been conducted in Western countries; research focusing on Chinese NMOSD patients—particularly considering China’s unique healthcare system, cultural context, and social support structure—remains limited. Furthermore, the majority of previous investigations have analyzed isolated factors rather than adopting an integrated biopsychosocial perspective. As a result, the relative contributions of modifiable factors such as sleep hygiene and psychological resilience remain poorly defined.

Therefore, this study aims to conduct a cross-sectional survey to evaluate the status of HRQoL among Chinese NMOSD patients. We systematically analyzed the variables across physiological (e.g., functional disability, sleep), psychological (e.g., depression, anxiety, resilience), and social (e.g., social support, alienation, social avoidance) domains. By elucidating these complex interactions, this study seeks to equip clinicians with a holistic understanding of patient vulnerabilities and to inform the development of multidimensional, culturally sensitive interventions tailored to the specific needs of Chinese individuals living with NMOSD.

## 2 Materials and methods

### 2.1 Design

This study conducted a cross-sectional design and was reported in accordance with the Strengthening of Reporting in Observational Studies in Epidemiology (STROBE) guidelines (20) (see Supplementary file S1).

### 2.2 Patients

Recruitment for this cross-sectional study occurred between October 10 and October 30, 2023, with the consecutive enrollment of NMOSD patients from the Guangdong Provincial Hospital of Traditional Chinese Medicine. Inclusion criteria were: (1) Diagnosis of neuromyelitis optica spectrum disorder (NMOSD) by a neurologist according to the 2015 International Panel (21) on NMO (IPND); (2) Age ≥ 18 years. Exclusion criteria were: (1) Pregnant or lactating women; (2) Concurrent severe chronic or acute illnesses (e.g., malignancy, severe hepatic/renal impairment, cardiovascular disease); (3) Confirmed pre-existing severe psychiatric disorders, dementia, or cognitive/language barriers resulting in an inability to communicate or complete the questionnaires reliably (22).

### 2.3 Sample size

This study incorporates 24 predictors and employs multiple linear regression as the primary analytical method. To ensure sufficient statistical power and reliability of the research findings, sample size calculation will primarily rely on a scientific power-based approach, supplemented by classical empirical guidelines. Using G*Power 3.1 software for estimation, with Cohen’s *f*^2^ as the effect size indicator, the parameters were set as follows: *α* = 0.05 (two-tailed), statistical power (1 − *β*) = 0.80, effect size *f*^2^ = 0.10, and number of predictors = 24. Based on these parameters, the minimum required effective sample size is approximately 176 participants. Considering potential issues such as invalid questionnaires, missing scale items, and non-response during the survey, a 10% non-response rate was applied for adjustment, resulting in an adjusted total sample size of 195. Furthermore, according to classical statistical theory, the sample size for multiple linear regression analysis should be 5–10 times the number of independent variables, yielding a recommended range of 120–240 participants for this study. Therefore, this study plans to include 180 participants. Ultimately, 216 eligible subjects completed the study, well exceeding the required threshold and fulfilling the classical empirical guideline.

### 2.4 Ethics approval

The Institutional Review Board of the Second Affiliated Hospital of Guangzhou University of Chinese Medicine approved this study (No. BE2023-096-01). All participants provided electronic informed consent prior to initiating the survey.

### 2.5 Instruments

This study did not aim to psychometrically validate these instruments; rather, we utilized their previously validated Chinese versions to comprehensively assess the biopsychosocial status of our target cohort. The Short-Form 36 Health Survey (SF-36) comprises 36 items, yielding two summary scores: the Physical Component Summary (PCS) and the Mental Component Summary (MCS). It also assesses eight health domains, including physical functioning, role limitations due to physical health, bodily pain, general health perceptions, vitality, social functioning, role limitations due to emotional problems, and mental health (23). The SF-36 offers a comprehensive assessment of health-related quality of life, with higher scores indicating better perceived health and well-being.

The basic activities of daily living (BADL) scale evaluates an individual’s functional independence in core self-care activities, such as eating, dressing, toileting, transferring, ambulation, and bathing. Higher BADL scores reflect greater independence in daily living and self-care abilities (24).

The Generalized Anxiety Disorder-7 (GAD-7) questionnaire consists of 7 items, with total scores ranging from 0 to 21 (25). Higher scores indicate more severe anxiety symptoms, and a cutoff score of ≥10 is generally regarded as indicative of clinically significant anxiety. The Cronbach’s *α* value for the GAD-7 in this study was 0.936.

The Patient Health Questionnaire-9 (PHQ-9) includes 9 items, with total scores ranging from 0 to 27 (26). Higher scores denote more severe depressive symptoms, and a score of ≥10 is typically used to define clinically significant depression.

The General Alienation Scale (GAS) measures an individual’s sense of alienation, reflecting subjective feelings of social isolation, meaninglessness, or loneliness. It assesses alienation across personal, social, and occupational contexts, with higher scores indicating stronger feelings of disconnection and existential distress (27).

The Social Avoidance and Distress Scale (SAD) is widely used in the assessment and research of social anxiety disorder. It specifically evaluates avoidance behaviors and subjective distress in social situations, with higher scores indicating greater social avoidance and interpersonal discomfort (28, 29).

The Perceived Social Support Scale (PSSS) assesses an individual’s subjective perception of social support from family, friends, and other significant sources, measuring the degree of satisfaction with and availability of support. Higher PSSS scores indicate stronger perceived social support (30).

The Connor-Davidson Resilience Scale (CD-RISC) evaluates multiple dimensions of psychological resilience, including adaptability, tolerance of negative affect, acceptance of change, sense of control, and spiritual influences. Comprising 25 items with a total score ranging from 0 to 100, it measures an individual’s capacity to adapt and recover from adversity, trauma, or stress (31). Higher scores indicate greater resilience. In the present study, the Cronbach’s *α* value of the CD-RISC was 0.962.

The Pittsburgh Sleep Quality Index (PSQI) assesses sleep quality across seven components: subjective sleep quality, sleep latency, sleep duration, sleep efficiency, sleep disturbances, use of sleep medication, and daytime dysfunction. Total scores range from 0 to 21, with higher scores indicating poorer sleep quality (32). A PSQI score >5 is generally considered indicative of poor sleep quality.

### 2.6 Data collection

Data collection comprised two distinct components to ensure clinical accuracy and objective reporting. Demographic characteristics and patient-reported outcomes (PROs) were collected via an online survey distributed through the Wenjuanxing platform. Demographic data included age, gender, educational level, marital status, employment status, living environment, and income level. Simultaneously, objective clinical variables—including disease duration, number of relapses, AQP4-IgG serostatus, current medication profile, and the Expanded Disability Status Scale (EDSS) score—were meticulously extracted from the hospital’s electronic medical records or evaluated by attending neurologists during clinical visits to prevent self-reporting bias. The self-reported scales consisted of the SF-36, BADL, GAD-7, PHQ-9, GAS, SAD, PSSS, CD-RISC, and PSQI.

### 2.7 Statistical analysis

All statistical analyses were performed using IBM SPSS Statistics for Windows, Version 27.0 (SPSS Inc., Chicago, IL, United States). The normality of continuous variables was assessed using the Kolmogorov–Smirnov test. Continuous variables that followed a normal distribution were presented as mean ± standard deviation (SD), whereas those that did not conform to a normal distribution were expressed as median (interquartile range, IQR). Categorical variables are presented as frequencies and percentages. Health-related quality of life was evaluated using the Physical Component Summary (PCS) and Mental Component Summary (MCS) of the SF-36. Spearman’s rank correlation analysis or independent samples *t*-test was used to investigate the associations of various influencing factors with PCS and MCS. Variables showing a significant correlation (*p* < 0.05) were subsequently entered into a multiple linear regression model using a stepwise selection procedure. Entry and exit criteria were set at *p* < 0.05 and *p* > 0.10, respectively, to identify the independent determinants of both the PCS and MCS. Variance Inflation Factor (VIF) and tolerance values were examined to ensure the absence of severe multicollinearity among the independent variables. All statistical tests were two-tailed, and statistical significance was defined as *p* < 0.05. To confirm the robustness of the model, the enter method was also performed, yielding consistent results (see Supplementary material for details).

## 3 Results

### 3.1 Patient characteristics and quality of life scores

A total of 216 NMOSD patients were finally enrolled in this study. The demographic and clinical characteristics of the participants are summarized in Table 1. The age of the patients ranged from 18 to 70 years (median of 37 years, IQR: 28–45), with a median disease duration of 50.5 months (IQR: 24–94). Among them, there was a female predominance (85.2%) and most patients (71.8%) were seropositive for AQP4-IgG. The cohort exhibited a moderate level of neurological disability, with a median Expanded Disability Status Scale (EDSS) score of 3.0 (IQR: 2.0–4.0). All patients (100%) were on immunosuppressant therapy, and a majority (59.3%) were exposed to polypharmacy (concurrent use of ≥5 drugs).

**Table 1:** Characteristics of study subjects (*n* = 216).

Patient characteristics	Total	PCS		MCS	
		*r*	*p*	*r*	*p*
Age, year (IQR)	37 (28–45)	−0.24	<0.001^*^	−0.054	0.433
Sex, *n* (%)		−0.06	0.382	−0.068	0.323
Female	184 (85.2)				
Male	32 (14.8)				
Educational level, *n* (%)		0.246	<0.001^*^	0.169	0.013^*^
Primary school or lower	7 (3.2)				
Lower secondary	49 (22.7)				
Upper secondary	42 (19.4)				
College or above	118 (54.6)				
Employment status, *n* (%)		−0.018	0.795	0.037	0.586
Unemployed	52 (24.1)				
Cognitively demanding work	104 (48.1)				
Physically demanding work	60 (27.8)				
Marital status, *n* (%)		−0.079	0.246	−0.076	0.265
Single	36 (16.7)				
Married	153 (70.8)				
Divorced	23 (10.6)				
Widowed	4 (1.9)				
Household, *n* (%)		0.043	0.534	−0.004	0.957
Live alone	27 (12.5)				
Live with family	178 (82.4)				
Others	11 (5.1)				
Activities of daily living, *n* (%)		−0.008	0.908	0.051	0.452
Independent	110 (50.9)				
Requires assistance	106 (49.1)				
Medical expenses payment method, *n* (%)		0.046	0.505	−0.057	0.401
Socialized medicine	1 (0.5)				
UEBMI	113 (52.3)				
URBMI	27 (12.5)				
NRCMS	66 (30.6)				
Out-of-pocket	9 (4.2)				
Income, RMB/mo, *n* (%)		0.162	0.017^*^	0.194	0.004^*^
No	94 (43.5)				
<3,000	41 (19.0)				
3,000–6,000	60 (27.8)				
6,000–10,000	15 (6.9)				
>10,000	6 (2.8)				
Disease duration, month (IQR)	50.5 (24–94)	−0.094	0.17	−0.102	0.136
Number of relapses (IQR)	2 (1–4)	−0.012	0.857	0.013	0.845
AQP4-IgG serostatus, *n* (%)		0.057	0.402	0.086	0.21
Positive	155 (71.8)				
Negative	61 (28.9)				
EDSS score (IQR)	3.0 (2.0–4.0)	0.001	0.994	0.018	0.793
Polypharmacy (≥5 drugs), *n* (%)	128 (59.3)	−0.134	0.049^*^	−0.12	0.078
Smoking history, *n* (%)	24 (11.1)	−0.089	0.195	−0.144	0.035^*^
Alcohol drinking history, *n* (%)	24 (11.1)	0.007	0.921	−0.006	0.934

PCS, physical component summary; MCS, mental component summary; IQR, interquartile range; UEBMI, urban employee-based basic medical insurance; URBMI, urban resident-based basic medical insurance; NRCMS, new rural cooperative medical scheme. Bold values and values marked with an asterisk (*) both indicate statistical significance at *p* < 0.05.

The HRQoL scores of the 216 NMOSD patients, assessed via the SF-36 scale, are summarized in Table 2. The median score of the PCS was 55.5 (IQR: 39.25–76.75), while the median score of the MCS was 51.0 (IQR: 34.25–73.25). Notably, the scores for Role-physical and Role-emotional domains were particularly low, with median scores of 25.00 and 33.33, respectively. These results indicate significant difficulties in both physical role functioning (e.g., fulfilling work/daily roles due to physical issues) and emotional role functioning (e.g., maintaining roles due to emotional distress), and the wide IQR also reflects large individual differences in these domains. In contrast, physical functioning scores were relatively higher (median: 80 IQR: 60–93.75).

**Table 2:** QoL scores in NMOSD patients (*n* = 216).

Scale	Scores
Physical functioning, median (IQR)	80 (60–93.75)
Role-physical, median (IQR)	25 (0–100)
Bodily pain, median (IQR)	62 (51–84)
General health, median (IQR)	45 (30–62)
Vitality, median (IQR)	50 (40–68.75)
Role-emotional, median (IQR)	33.33 (0–100)
Social functioning, median (IQR)	66.67 (44.44–77.78)
Mental health, median (IQR)	52 (44–68)
PCS, median (IQR)	55.5 (39.25–76.75)
MCS, median (IQR)	51 (34.25–73.25)

PCS, physical component summary; MCS, mental component summary; IQR, interquartile range.

### 3.2 Reliability of measurement instruments

To verify the internal consistency of the psychosocial and functional scales applied within our specific Chinese NMOSD cohort, Cronbach’s alpha (*α*) coefficients were calculated. In the current cohort, all scales demonstrated excellent internal consistency and reliability, fully meeting the requirements for clinical evaluation: SF-36 (*α* = 0.924), BADL (*α* = 0.924), GAD-7 (*α* = 0.936), PHQ-9 (*α* = 0.931), GAS (*α* = 0.891), SAD (*α* = 0.870), PSSS (*α* = 0.949), CD-RISC (*α* = 0.962), and PSQI (*α* = 0.871).

### 3.3 Univariate associations with physical and mental HRQoL

Bivariate analyses were conducted to examine factors associated with the PCS and MCS scores, as summarized in Tables 1 and 3.

**Table 3:** Self-reported questionnaires for NMOSD patients (*n* = 216).

Scales	Scores	PCS		MCS	
		*r*	*p*	*r*	*p*
BADL, median (IQR)	100 (95–100)	0.386	**<0.001** ^*^	0.299	**<0.001** ^*^
GAD-7, median (IQR)	7 (4–11)	−0.407	**<0.001** ^*^	−0.631	**<0.001** ^*^
PHQ-9, median (IQR)	9 (4–14)	−0.52	**<0.001** ^*^	−0.72	**<0.001** ^*^
GAS, mean (SD)	35.98 ± 7.23	−0.425	**<0.001** ^*^	−0.577	**<0.001** ^*^
SAD, median (IQR)	7 (4–11)	−0.325	**<0.001** ^*^	−0.473	**<0.001** ^*^
PSSS, median (IQR)	62 (50–71)	0.237	**<0.001** ^*^	0.344	**<0.001** ^*^
CD-RISC, mean (SD)	55.56 ± 18.20	0.333	**<0.001** ^*^	0.456	**<0.001** ^*^
PSQI, median (IQR)	8 (5–12)	−0.515	**<0.001** ^*^	−0.561	**<0.001** ^*^

SD, standard deviation; BADL, basic activities of daily living; GAD-7, Generalized Anxiety Disorder-7 questionnaire; PHQ-9, Patient Health Questionnaire-9; GAS, General Alienation Scale; SAD, Social Avoidance and Distress Scale; PSSS, Perceived Social Support Scale; CD-RISC, Connor-Davidson Resilience Scale; PSQI, Pittsburgh Sleep Quality Index. Bold values and values marked with an asterisk (*) both indicate statistical significance at *p* < 0.05.

Among demographic and clinical variables (Table 1), a higher educational level was positively correlated with PCS (*r* = 0.246, *p* < 0.001) and MCS (*r* = 0.169, *p* = 0.013). Similarly, higher monthly income was positively associated with both PCS (*r* = 0.162, *p* = 0.017) and MCS (*r* = 0.194, *p* = 0.004). In contrast, older age (*r* = −0.24, *p* < 0.001) and polypharmacy (*r* = −0.134, *p* = 0.049) were negatively correlated with PCS. Smoking history was inversely associated with MCS (*r* = −0.144, *p* = 0.035). No other demographic or disease-specific characteristics—including EDSS score, relapse history, AQP4-IgG serostatus, disease duration, sex, employment, marital status, living situation, independence in daily activities, or payment method—were significantly correlated with PCS or MCS (*p* > 0.05).

Analysis of self-reported psychosocial and functional measures (Table 3) revealed that better performance in basic activities of daily living (BADL) was strongly associated with higher PCS (*r* = 0.386, *p* < 0.001) and MCS (*r* = 0.299, *p* < 0.001). Greater perceived social support (PSSS) and higher psychological resilience (CD-RISC) were also positively correlated with both PCS (PSSS: *r* = 0.237, *p* < 0.001; CD-RISC: *r* = 0.333, *p* < 0.001) and MCS (PSSS: *r* = 0.344, *p* < 0.001; CD-RISC: *r* = 0.456, *p* < 0.001). Conversely, more severe depressive symptoms (PHQ-9), anxiety (GAD-7), general alienation (GAS), social avoidance and distress (SAD), and poorer sleep quality (PSQI) demonstrated significant inverse correlations with both PCS and MCS (all *p* < 0.001). It is noteworthy that depressive symptoms exhibited the strongest negative correlation with both mental health and physical health (MCS: *r* = −0.72; PCS: *r* = −0.52).

### 3.4 Independent associated factors of physical and mental HRQoL

Variables that demonstrated statistical significance (*p* < 0.05) in the univariate analyses were incorporated into separate multiple stepwise linear regression models to identify independent associated factors of PCS and MCS. All tolerance values exceeded 0.5 and variance inflation factors (VIFs) were below 2, suggesting no multicollinearity concerns.

As detailed in Table 4, the regression model for PCS was statistically significant (*F* = 29.961, *p* < 0.001), explaining 40.2% of the variance (Adjusted *R*^2^ = 0.402). Older age (*β* = −0.194, 95% CI: −0.606 to −0.167), more severe depressive symptoms (PHQ-9; *β* = −0.290, 95% CI: −1.438 to −0.505), higher levels of social avoidance and distress (SAD; *β* = −0.128, 95% CI: −1.320 to −0.033), and poorer sleep quality (PSQI; *β* = −0.282, 95% CI: −2.082 to −0.782) emerged as independent predictors of worse physical HRQoL. In contrast, better functional independence (BADL) was an independent predictor of better PCS (*β* = 0.132, 95% CI: 0.034 to 0.292).

**Table 4:** Multiple stepwise linear regressions for predictive variables of physical component summary (*n* = 216).

Variables	*B*	SE	*β* (95% CI)	*t*	*p*	Tolerance	VIF
Constant	83.194	8.084		10.291	**<0.001**		
Age	−0.386	0.111	−0.194 (−0.606, −0.167)	−3.471	**0.001**	0.888	1.126
BADL	0.163	0.065	0.132 (0.034, 0.292)	2.486	**0.014**	0.986	1.014
PHQ-9	−0.972	0.237	−0.29 (−1.438, −0.505)	−4.108	**<0.001**	0.558	1.793
SAD	−0.677	0.326	−0.128 (−1.32, −0.033)	−2.074	**0.039**	0.726	1.377
PSQI	−1.432	0.33	−0.282 (−2.082, −0.782)	−4.342	**<0.001**	0.657	1.521

*R*^2^ = 0.416, adjusted *R*^2^ = 0.402, *F* = 29.961, *p* < 0.001; *B*, unstandardized coefficient; SE, standard error; *β*, standardized coefficient beta; CI, confidence interval; VIF, variance inflation factor. Bold values and values marked with an asterisk (*) both indicate statistical significance at *p* < 0.05.

The regression model for MCS was also highly significant (*F* = 73.928, *p* < 0.001), accounting for 57.6% of the variance (Adjusted *R*^2^ = 0.576), as presented in Table 5. More severe depressive symptoms (PHQ-9; *β* = −0.439, 95% CI: −1.879 to −1.089), greater social avoidance and distress (SAD; *β* = −0.135, 95% CI: −1.316 to −0.124), and poorer sleep quality (PSQI; *β* = −0.267, 95% CI: −1.897 to −0.837) were identified as independent predictors of poorer mental HRQoL. Higher psychological resilience (CD-RISC) was an independent predictor of better MCS (*β* = 0.127, 95% CI: 0.026 to 0.287).

**Table 5:** Multiple stepwise linear regressions for predictive variables of mental component summary (*n* = 216).

Variables	*B*	SE	*β* (95% CI)	*t*	*p*	Tolerance	VIF
Constant	76.738	5.699		13.466	**<0.001**		
PHQ-9	−1.484	0.201	−0.439 (−1.879, −1.089)	−7.398	**<0.001**	0.561	1.784
SAD	−0.72	0.302	−0.135 (−1.316, −0.124)	−2.383	**0.018**	0.612	1.634
CD-RISC	0.156	0.066	0.127 (0.026, 0.287)	2.357	**0.019**	0.684	1.462
PSQI	−1.367	0.269	−0.267 (−1.897, −0.837)	−5.086	**<0.001**	0.714	1.4

*R*^2^ = 0.584, adjusted *R*^2^ = 0.576, *F* = 73.928, *p* < 0.001; *B*, unstandardized coefficient; SE, standard error; *β*, standardized coefficient beta; CI, confidence interval; VIF, variance inflation factor. Bold values and values marked with an asterisk (*) both indicate statistical significance at *p* < 0.05.

## 4 Discussion

This cross-sectional study systematically evaluated HRQoL and its influencing factors in 216 Chinese patients with NMOSD. Our results showed that Chinese NMOSD patients experience severe HRQoL impairments, with both physical and mental health components significantly compromised. Notably, the most severely affected domains were Role-Physical and Role-Emotional, highlighting specific vulnerabilities in maintaining physical role functioning and emotional societal roles. Critically, multivariate analysis revealed that psychosocial factors—particularly depressive symptoms and poor sleep quality, and social avoidance—are the strongest independent predictors of HRQoL. This finding emphasizes the need to shift from a purely biomedical model to an integrated biopsychosocial framework when evaluating patient outcomes in NMOSD. Thus, this study provides a large-scale, multidimensional characterization of factors influencing HRQoL in a Chinese NMOSD cohort, offering robust evidence to inform the development of culturally and clinically tailored multidimensional intervention strategies within the Chinese context.

In this study, the PCS and MCS scores (55.5 and 51.0, respectively) in Chinese NMOSD patients were significantly lower than those of the general Chinese population (PCS: 79.36, MCS: 77.00) (33), indicating comprehensive impairments consistent with other research findings (6, 16, 34, 35). Compared with international epidemiological data, the pattern of QoL impairment in Chinese NMOSD patients shares similarities with global trends (e.g., high female predominance, severe impact on mental health) but also exhibits population-specific characteristics. For instance, the female-to-male ratio in this study was 5.7:1, slightly lower than the global ratio of 8-9:1 (36), suggesting a modestly broader male representation in the Chinese cohort.

Crucially, this study identified severely diminished scores in the Role-Physical (median: 25) and Role-Emotional (median: 33.33) domains of the SF-36 among Chinese NMOSD patients, indicating a more pronounced impairment in these areas compared to Western cohorts, where median scores typically range between 40 and 50 (34). This discrepancy stems from a complex interplay of disease-related, psychological, social, and cultural factors. From a clinical perspective, the high relapse risk inherent to NMOSD, coupled with the fatigue, cognitive side effects, and emotional disturbances associated with long-term polypharmacy, directly undermines the physical and mental stamina and stability required for patients to consistently fulfill their societal roles. The most critical sociocultural mechanism lies in the heightened expectations and rigid demands within Chinese society regarding family roles (e.g., supporting aging parents, raising children) and occupational roles (which emphasize stability and full attendance). When disease progression leads to fluctuations or interruptions in a patient’s capacity, it often results in profound feelings of guilt, shame, and a sense of being stripped of their social identity. Furthermore, the relatively underdeveloped psychosocial support system in China means that emotional distress often goes unaddressed through professional channels, thereby exacerbating difficulties in role adaptation (37). This stark contradiction between preserved basic physical functioning and collapsed social roles highlights the urgent need for targeted role-function support. Depressive symptoms emerged as the strongest independent negative predictor of MCS and also served as a key predictor of PCS, aligning with previous findings (38). The relationship between depression and NMOSD is bidirectional: disease unpredictability and fear of disability trigger depression, while depression impairs self-management and amplifies physical discomfort, further deteriorating QoL. Notably, unlike some Western studies (15), anxiety did not remain an independent predictor in our multivariate model. This likely reflects the high anxiety-depression comorbidity in Chinese patients, suggesting that depression mediates anxiety’s impact. The affective-cognitive symptoms of depression may exert a more central influence on long-term life satisfaction than somatic anxiety.

Sleep quality (PSQI) was identified as a robust independent predictor of both physical health and mental health in NMOSD patients, underscoring its dual detrimental effect on HRQoL. This finding is particularly significant, as sleep disturbances are often simplistically attributed to pain or medication side effects. The underlying pathophysiology is multidimensional. On the physical front, chronic sleep deprivation profoundly impedes motor recovery, exacerbates fatigue, and lowers pain tolerance, explaining its exceptionally strong negative correlation with PCS (*r* = −0.515) found in our cohort. On the mental front, poor sleep continuity directly impairs emotional regulation, serving as a catalyst for cognitive fatigue and exacerbating depressive symptoms. Furthermore, the etiology of sleep disturbance in NMOSD is complex: it reflects a combination of primary neural injury (as AQP4 antibodies can directly target sleep-regulating centers like the hypothalamus), secondary physical symptoms (e.g., neuropathic pain, nocturia), and the disruption of sleep architecture by polypharmacy (e.g., corticosteroids). In this study, the median PSQI score was 8, suggesting that over half of the patients experienced significant sleep disturbances; however, sleep is rarely targeted as an intervention in NMOSD clinical management. Unlike the EDSS, which captures static disability, sleep quality is a dynamic and modifiable factor that may serve as an early warning indicator for HRQoL deterioration.

Social Avoidance and Distress (SAD) emerged as an independent predictor of both PCS and MCS, while the General Sense of Alienation (GAS) showed strong univariate correlations but did not enter the final regression models. This reveals the unique mechanism by which impaired social connectedness impacts quality of life in patients with NMOSD. Social avoidance stems primarily from fear of disease-related stigma and internalization of a disabled identity, presenting as a protective behavioral withdrawal that reinforces a self-perpetuating cycle of isolation. This construct delineates a complex pathway of disease-psychosocial interaction: social avoidance erodes social support systems, impairs the perception of physical function, and exacerbates psychological suffering through self-isolating behaviors. Alongside these behavioral and psychosocial challenges, demographic vulnerabilities also significantly dictate physical outcomes. Specifically, older age emerged as an independent predictor of poorer Physical Component Summary (PCS) scores. This association may be attributed to age-related declines in physiological reserve and resilience, potentially rendering older patients more vulnerable to neurological damage. Consistent with previous clinical observations (39, 40), aging or late-onset disease predisposes patients to a substantially higher risk of structural motor disability, which directly translates to diminished physical quality of life.

Beyond psychological factors, socioeconomic and demographic variables may also shape patients’ quality-of-life experiences. In the univariate analysis, higher educational attainment and monthly income were associated with better QoL, suggesting that socioeconomic advantage may confer both improved access to healthcare resources and greater protection against the cumulative financial burden of long-term NMOSD management. By contrast, objective household status (i.e., living alone versus living with family) was not significantly associated with either PCS or MCS (*p* > 0.05). This finding indicates that structural cohabitation alone does not necessarily translate into meaningful support. Rather, it is the perceived quality of interpersonal relationships and subjective social support—as reflected by the PSSS—that may be more relevant to patients’ well-being. Although education and income did not remain in the final multivariate models, their effects may be indirect, operating through downstream pathways such as resilience, sleep quality, and access to supportive care.

Against this broader psychosocial backdrop, one of the most noteworthy findings of the present study is that conventional disease-specific indicators—including EDSS score, relapse frequency, and disease duration—did not independently predict HRQoL in the multivariate analyses. Rather than representing an anomalous result, this pattern may reflect the clinical and social realities of the contemporary Chinese NMOSD population. First, our cohort had a median EDSS score of 3.0, and all patients were receiving immunosuppressive therapy, indicating a group with moderate but relatively stable disability. In this context, as modern treatment increasingly controls acute inflammatory activity and limits overt neurological deterioration, the determinants of QoL may shift from disease severity per se toward the long-term psychosocial consequences of living with a chronic disabling condition. Second, disease burden may affect QoL indirectly through psychosocial pathways. Recurrent relapses and accumulated disability can precipitate depression, sleep disturbance, and social avoidance; once these more proximal determinants are entered into multivariate models, the direct contribution of clinical variables may no longer remain significant. Third, this finding underscores an inherent limitation of the EDSS, which places disproportionate emphasis on ambulation. In NMOSD, disability is often driven by visual impairment due to severe optic neuritis; accordingly, some patients may retain walking ability and therefore have relatively low EDSS scores, despite experiencing marked loss of independence and substantial deterioration in overall QoL. This may explain why BADL, which more directly captures essential self-care and day-to-day functioning, showed greater practical relevance in reflecting the lived burden of NMOSD.

Psychological resilience (CD-RISC) emerged as an independent positive predictor of MCS, underscoring its buffering role in mental health. Patients with higher resilience demonstrated enhanced capacity to adapt to disease-related challenges, regulate negative emotions, and maintain a sense of control. Within the Chinese collectivist context, resilience may be bolstered by strong family connections. However, our cohort’s average resilience score was lower than the national normative value (41), indicating significant potential for improving patient outcomes through targeted resilience-building interventions.

Based on these findings, we advocate for multidimensional and culturally sensitive interventions targeting mental health, sleep, and social functioning, thereby providing actionable insights for improving patients’ overall quality of life. To operationalize this approach, the comprehensive management of NMOSD can be optimized through several specific strategies. First, routine follow-up should integrate mental health and sleep assessments, allowing for prompt specialist referrals (e.g., psychiatry or sleep medicine) when clinically significant symptoms are identified. Second, psychological resilience training should be strengthened by developing culturally adapted resilience-building programs—such as mindfulness-based stress reduction and peer support groups—to enhance patients’ coping skills (42). Finally, to mitigate social avoidance, psychosocial support systems could be strengthened through public education, patient networks, and accessible social or employment support, thereby effectively addressing patients’ unmet social needs.

This study has several limitations that should be acknowledged. First, the cross-sectional design precludes causal inference, and no clear distinction was made between patients with recent relapse and those in remission. Second, the single-center sample may limit the generalizability of our findings to other regions in China, given variations in healthcare accessibility and cultural practices across provinces. Third, data collection relied on self-reported measures, which are susceptible to recall bias and common method bias (e.g., social desirability bias when reporting social support or resilience). Fourth, the absence of objective measures (e.g., objective sleep monitoring, neuroimaging data) limited our ability to validate self-reported outcomes. Future research should involve large-scale, multi-center longitudinal studies to track quality of life over time and establish causal relationships between predictors and outcomes. Integrating objective measures (e.g., polysomnography for sleep quality, MRI) with self-reported data would also provide a more comprehensive assessment.

## 5 Conclusion

In summary, Chinese NMOSD patients experience profound HRQoL impairment, with the most severe deficits in Role-physical and Role-Emotional domains. Psychosocial factors—such as depression, poor sleep quality, psychological resilience, and social avoidance—together with functional independence (BADL) and older age play a more pivotal role than traditional clinical measures. These findings underscore the necessity for multidimensional and culturally informed interventions to address the complex biopsychosocial needs of this patient population. By adopting a patient-centered care model grounded in the biopsychosocial framework, clinicians and policymakers can work toward improving long-term outcomes and enhancing the quality of life for individuals living with NMOSD in China.

## Data Availability

The raw data supporting the conclusions of this article will be made available by the authors, without undue reservation.
