# Supplementary material for: Quality of life and its influencing factors in patients with neuromyelitis optica spectrum disorder
Source: Front Neurol. 2026 Jun 19;17:1797747. doi: 10.3389/fneur.2026.1797747 (PMC13327940; doi:10.3389/fneur.2026.1797747)
Supplement: Supplementary file 2 [file Table_1.docx]

**Statistical analysis**

Variables demonstrating significant correlation (P < 0.05) in univariate analysis, along with Disease duration, Number of relapses, and EDSS which were included based on clinical requirements, were entered into a multiple linear regression model using the Enter method to identify independent determinants of the PCS and MCS. All statistical analyses were two-tailed, with a P-value < 0.05 considered statistically significant.

**Results**

The results of the multiple linear regression model using the Enter method indicated that PHQ-9, CD-RISC, and PSQI were independent predictors of MCS, while BADL, PHQ-9, PSQI, and Age were independent predictors of PCS. These findings were largely consistent with those obtained via the stepwise method, demonstrating the robustness and reliability of the model. Therefore, the stepwise method was employed to build the regression model in the main text.

**Table 1. Multiple Linear Regressions For associated Variables Of Mental Component Summary (n=216)**

| Variables | B | SE | β(95%CI) | t | P | Tolerance | VIF |
| --- | --- | --- | --- | --- | --- | --- | --- |
| Constant | 67.087 | 13.267 |  | 5.057 | <0.001 |  |  |
| Disease duratio | 0.009 | 0.018 | 0.009(-5.741,2.897) | -0.499 | 0.618 | 0.86 | 1.162 |
| Polypharmacy | -1.422 | 2.19 | -1.422(-5.741,2.897) | -0.649 | 0.517 | 0.856 | 1.168 |
| Number of relapses | 0.222 | 0.445 | 0.222(-0.655,1.099) | 0.499 | 0.618 | 0.855 | 1.17 |
| EDSS | -0.468 | 0.628 | -0.468(-1.708,0.771) | -0.745 | 0.457 | 0.844 | 1.185 |
| BADL | 0.097 | 0.058 | 0.097(-0.018,0.211) | 1.664 | 0.098 | 0.908 | 1.102 |
| GDA-7 | -0.338 | 0.354 | -0.338(-1.037,0.361) | -0.953 | 0.342 | 0.3 | 3.336 |
| **PHQ-9** | -1.164 | 0.307 | -1.164(-1.770,-0.557) | -3.786 | **<0.001** | 0.239 | 4.183 |
| GSA | -0.165 | 0.213 | -0.165(-0.585,0.255) | -0.774 | 0.440 | 0.419 | 2.385 |
| SAD | -0.585 | 0.336 | -0.585(-1.246,0.077) | -1.743 | 0.083 | 0.497 | 2.012 |
| PSSS | -0.009 | 0.086 | -0.009(-0.179,0.161) | -0.104 | 0.918 | 0.589 | 1.699 |
| **CD-RISC** | 0.177 | 0.075 | 0.177(0.029,0.326) | 2.353 | **0.020** | 0.529 | 1.891 |
| **PSQI** | -1.281 | 0.314 | -1.281(-1.901,-0.661) | -4.075 | **<0.001** | 0.523 | 1.91 |
| Educational level |  |  |  |  |  |  |  |
| Lower secondary | 11.198 | 6.193 | 11.198(-1.017,23.413) | 1.808 | 0.072 | 0.147 | 6.791 |
| Upper secondary | 5.42 | 6.557 | 5.420(-7.512,18.352) | 0.827 | 0.409 | 0.147 | 6.798 |
| College or above | 5.131 | 6.525 | 5.131(-7.738,17.999) | 0.786 | 0.433 | 0.094 | 10.652 |
| Age | -0.104 | 0.108 | -0.104(-0.318,0.110) | -0.962 | 0.337 | 0.677 | 1.476 |
| Smoking History | -1.689 | 2.365 | -1.689(-6.353,2.975) | -0.714 | 0.476 | 0.787 | 1.271 |
| Income |  |  |  |  |  |  |  |
| <3000 | 4.467 | 2.971 | 4.467(-1.392,10.326) | 1.504 | 0.134 | 0.73 | 1.37 |
| 3000-6000 | 4.352 | 2.819 | 4.352(-1.208,9.912) | 1.544 | 0.124 | 0.621 | 1.609 |
| 6000-10000 | 3.53 | 4.439 | 3.530(-5.225,12.285) | 0.795 | 0.427 | 0.778 | 1.285 |
| >10000 | 11.39 | 6.721 | 11.390(-1.866,24.646) | 1.695 | 0.092 | 0.812 | 1.232 |

Notes: R^2^=0.617, adjustedR^2^=0.575, F=14.861, P<0.001; B= unstandardized coefficient; SE = standard error; β = standardized coefficient Beta; CI = Confidence Interval; VIF = variance inflation factor.

**Table 2. Multiple Linear Regressions For Associated Variables Of Physical Component Summary (n=216)**

| **Variables** | **B** | **SE** | **β(95%CI)** | **t** | **P** | **Tolerance** | **VIF** |
| --- | --- | --- | --- | --- | --- | --- | --- |
| Constant | 81.343 | 15.746 |  | 5.166 | **<0.001** |  |  |
| Disease duratio | -0.009 | 0.021 | -0.025(-0.051,0.033) | -0.435 | 0.664 | 0.866 | 1.154 |
| Polypharmacy | -3.134 | 2.591 | -0.069(-8.245,1.977) | -1.209 | 0.228 | 0.865 | 1.156 |
| Number of relapses | 0.038 | 0.528 | 0.004(-1.003,1.08) | 0.073 | 0.942 | 0.858 | 1.165 |
| EDSS | -0.428 | 0.747 | -0.033(-1.901,1.045) | -0.573 | 0.567 | 0.847 | 1.181 |
| **BADL** | 0.147 | 0.069 | 0.119(-0.011,0.282) | 2.131 | **0.034** | 0.913 | 1.095 |
| GDA-7 | 0.384 | 0.421 | 0.089(-0.446,1.215) | 0.913 | 0.363 | 0.301 | 3.327 |
| **PHQ-9** | -1.042 | 0.365 | -0.311(-1.762,-0.322) | -2.853 | **0.005** | 0.240 | 4.169 |
| GSA | -0.214 | 0.253 | -0.069(-0.712,0.285) | -0.845 | 0.399 | 0.422 | 2.372 |
| SAD | -0.389 | 0.397 | -0.074(-1.173,0.395) | -0.979 | 0.329 | 0.502 | 1.994 |
| PSSS | -0.123 | 0.103 | -0.084(-0.326,0.079) | -1.201 | 0.231 | 0.589 | 1.698 |
| CD-RISC | 0.147 | 0.089 | 0.12(-0.029,0.323) | 1.643 | 0.102 | 0.534 | 1.874 |
| **PSQI** | -1.355 | 0.363 | -0.267(-2.07,-0.64) | -3.738 | **<0.001** | 0.557 | 1.795 |
| Educational level |  |  |  |  |  |  |  |
| Lower secondary | 6.724 | 7.276 | 0.127(-7.625,21.073) | 0.924 | 0.357 | 0.151 | 6.617 |
| Upper secondary | 4.425 | 7.624 | 0.079(-10.61,19.46) | 0.580 | 0.562 | 0.154 | 6.489 |
| College or above | 7.216 | 7.498 | 0.162(-7.571,22.003) | 0.962 | 0.337 | 0.101 | 9.931 |
| **Age** | -0.365 | 0.125 | -0.183(-0.611,-0.118) | -2.912 | **0.004** | 0.719 | 1.390 |
| Income |  |  |  |  |  |  |  |
| <3000 | 2.337 | 3.532 | 0.041(-4.629,9.303) | 0.662 | 0.509 | 0.731 | 1.368 |
| 3000-6000 | 3.663 | 3.331 | 0.074(-2.907,10.233) | 1.100 | 0.273 | 0.630 | 1.587 |
| 6000-10000 | 4.617 | 5.245 | 0.053(-5.727,14.961) | 0.880 | 0.380 | 0.789 | 1.267 |
| >10000 | 2.642 | 7.939 | 0.02(-13.014,18.299) | 0.333 | 0.740 | 0.824 | 1.213 |

Notes: R^2^=0.667, adjustedR^2^=0.388, F=7.803, P<0.001; B= unstandardized coefficient; SE = standard error; β = standardized coefficient Beta; CI = Confidence Interval; VIF = variance inflation factor.
